# SowPostureDS: A Multi-Class Image Dataset for YOLO-Based Detection of Sow Postures in diverse Farrowing Systems

**DOI:** 10.1038/s41597-026-07788-x

**Published:** 2026-07-04

**Authors:** Johann Wahmhoff, Imke Traulsen, Astrid L. van Asten, Joachim Krieter, Neele Dirksen, Sophie Diers, Martin Wutke

**Affiliations:** 1 Institute of Animal Breeding and Husbandry, Christian-Albrechts-University Kiel, Hermann-Rodewald-Str. 6, 24118 Kiel, Germany; 2 Chamber of Agriculture of North Rhine-Westphalia, Centre for Agriculture Haus Düsse, Haus Düsse 2, 59505 Bad Sassendorf, Germany; 3 Chamber of Agriculture of Lower Saxony, Mars-la-Tour-Str. 6, 26121 Oldenburg, Germany; 4 Chamber of Agriculture of Schleswig-Holstein, Gutshof 1, 24327 Blekendorf, Germany

## Abstract

Accurate detection of sow postures provides valuable insights into animal activity, which is a key indicator for health, welfare, and productivity. While activity monitoring using wearable sensors has become a standard approach for certain livestock species like dairy cattle, its application in pig production remains limited due to practical challenges. Advances in computer vision (CV) and artificial intelligence (AI) offer an alternative, enabling non-invasive monitoring of pig behavior. However, the development of robust CV models requires large and diverse datasets that capture the variability of housing environments and visual conditions. To address this limitation, we present SowPostureDS, a dataset comprising 14,400 annotated images of sows from different farrowing systems. Images were extracted from long-term video recordings and manually annotated into four posture classes following a consistent scheme. The dataset reflects heterogeneity in housing design, lighting conditions, and the presence of piglets, providing a valuable resource for implementing CV models with improved robustness across diverse environments. A use case demonstrates the dataset’s applicability, showing that models trained on SowPostureDS can achieve high accuracy and can be effectively adapted to unseen environments using transfer learning with minimal additional data.

## Background & Summary

Monitoring of animal activity is a key component of many precision livestock farming systems, as activity patterns provide important indicators of health, welfare, and productivity^1,2^. In sows, changes in activity and posture can signal farrowing events, health disorders or compromised welfare conditions^3–5^. While activity monitoring is already widely applied in various livestock species, where pedometers and accelerometers are often used to detect estrus or lameness^6–9^, comparable approaches are far less common in pig production^10^. This is primarily a result of practical constraints, e.g. the relatively short production cycles in finishing pigs and the technical challenges of attaching and maintaining wearable sensors on the animals^11,12^. Devices fixed to the neck or legs are often lost or damaged, and their use can interfere with natural behavior^11,13^.

With rapid advances in CV, camera-based monitoring has emerged as a promising alternative. Vision-based methods are non-invasive, cost-efficient once installed, and suitable for continuous, long-term monitoring without physical contact with the animals^10^. Several studies have applied CV techniques, such as motion pattern analysis, pixel-level dynamics, or feature-based tracking algorithms, to estimate pig activity without explicit detection of pigs and postures^14,15^. However, these traditional CV approaches face limitations, as tracking multiple visually indistinguishable animals is challenging, and activity estimation based on pixel variations can be influenced by illumination changes or video quality^16,17^. To address these limitations, more sophisticated Deep Learning (DL) methods like object detection and posture classification have been applied to determine the location of a sow within an image and to classify its postures into a predefined category. Both the position of a sow within the pen and its body posture can serve as reliable indicators of activity levels and behavioral state. For example, Girardie *et al*. (2022) applied object detection to classify sow postures such as standing, sitting, ventral lying, and lateral lying, as well as activity-related behaviors like feeding and drinking^18^. By analyzing the temporal distribution of these postures, they identified activity patterns that were significantly associated with piglet survival and early growth, demonstrating how posture-based detection can provide interpretable and biologically meaningful indicators of sow activity. Similarly, Song *et al*. (2024) employed an object detection model based on the YOLOv5 model architecture to classify sow postures from continuous CCTV recordings^19^. The system successfully distinguished between key postures such as lying, standing, and sitting, providing detailed information on the temporal distribution of sow activity. These posture-based measures were then used to infer estrus-related behavioral changes, demonstrating the value of direct posture detection for monitoring reproductive status. Both works illustrate the potential of posture-based activity recognition but have so far relied on datasets, restricted to a single environment, and rarely made publicly available.

However, training robust and generalizable DL models requires large and heterogeneous datasets^20–22^. But creating datasets is challenging and labor-intensive, especially when environments vary widely, as in livestock husbandry. In pig production, housing systems differ substantially across farms in terms of flooring, pen design, enrichment, and lighting conditions^20^. These differences hinder the transferability of models trained in one farm environment to another, limiting the practical application of existing solutions. Moreover, publicly available datasets for sow posture detection remain scarce, posing a barrier to progress in this research domain.

To address this limitation, we propose SowPostureDS^23^, a dataset designed to support the development of robust CV and DL models for sow posture detection in farrowing environments. The dataset comprises annotated images collected across three distinct housing environments, with balanced representation of four posture classes, including both day- and nighttime recordings. By combining multiple environments into a single dataset, SowPostureDS provides diverse training data that improves model robustness and facilitates adaptation to new environments using transfer learning.

Beyond posture detection, SowPostureDS has the potential to support broader applications in sow monitoring, including activity recognition, welfare assessment, and reproductive management. By providing an openly available dataset, we aim to lower the entry barrier for researchers and practitioners in precision livestock farming and to accelerate progress toward practical, camera-based monitoring solutions. In line with the FAIR (**F**indability, **A**ccessibility, **I**nteroperability, and **R**eusability) data principles^24^, SowPostureDS contributes to the transparency, reproducibility, and accessibility of research data in animal science. We anticipate that this resource will not only enable the development of accurate and robust posture detection models but also foster new approaches for activity monitoring and welfare assessment in pig production.

## Methods

Given the wide variety of farrowing environments in modern pig production, it is essential for the training and application of robust computer vision models to incorporate a broad range of heterogeneous information, including different housing environments, lighting conditions, and video parameters. Therefore, the images included in the proposed dataset originate from three publicly funded German research projects: ZISSAU Phase 2 (funding code: 28N305603, project period: 12/19/2023–07/31/2026), DigiSchwein (funding code: 28DE109G18, project period: 02/10/2020–08/31/2024) and InnoPig (funding code: 2817205413, project period: 07/03/2015–02/28/2019). The Projects were conducted in distinct housing environments and over varying time periods. Since each project corresponds to an individual husbandry environment, three distinct environment names (Env A–C) are used in the following to increase readability.

### Overview of housing-systems and recording setups

A high-level comparison of key characteristics across the three housing environments is provided in Table 1. In Env A, data were recorded from October 2022 to September 2023 on a conventional farm in Northern-Germany. Data collection was carried out in 11 batches, with an average of 15 recorded sows. In Env B, data were collected from March 2021 to September 2023 at the Chamber of Agriculture of Lower Saxony in Wehnen, Germany. Recordings covered 23 batches, each involving eight sows. In Env C, data were recorded between April 2016 and January 2017 at the research farm Futterkamp (Chamber of Agriculture Schleswig-Holstein). Data collection included four batches with 20 sows each. In all environments, infrared (IR) lighting was used for nighttime recordings.

**Table 1:** Overview of the housing systems and recording setups across the three environments.

	Env A	Env B	Env C
Housing system	BeFree (Schauer Agrotronic GmbH^34^)	Kombi-Fix (EnSta Stalltechnik GmbH^35^)	Free-farrowing pens in a group-nursing system (Big Dutchman^36^)
Confinement	Temporary confinement	Temporary confinement	No confinement
Floor space (m²)	6.5	6.7	6.0
Camera type	RLC-520A (Reolink^37^)	M3206-LVE (Axis^38^)	M3024-LVE (Axis^38^)
Camera perspective	Top-down	Top-down	Slightly angled top-down
Camera height (m)	3.2	3.0	2.1–2.2
Resolution	2560 × 1920	1920 × 1080	1280 × 800
Frame rate (FPS)	30	20	5

### Image extraction, annotation, and dataset compilation

To generate a representative and diverse dataset, videos for image extraction were manually selected following a consistent procedure across all three projects. Selection criteria included a broad representation of different animals and pens, ensuring that all four defined postures were present and that both daytime and nighttime recordings were sufficiently covered for each posture. Additionally, an effort was made to include a balanced range of environmental conditions, such as variations in lighting or the presence or absence of piglets. From these selected videos, frames were extracted at specific intervals depending on the activity level of the sow. Frames were extracted at variable intervals ranging from multiple frames per second during high activity periods to approximately one frame per minute during low-activity periods.

To ensure consistency and reproducibility of the annotation process, all images were labeled following a standardized annotation protocol. Annotation was performed using the LabelMe software^25^ (version 5.2.1), where each image was assigned a single bounding box enclosing the focal sow. Only sows located in the focal pen have been annotated, while partially visible animals from neighboring pens have not been taken into account.

**Fig. 1:**
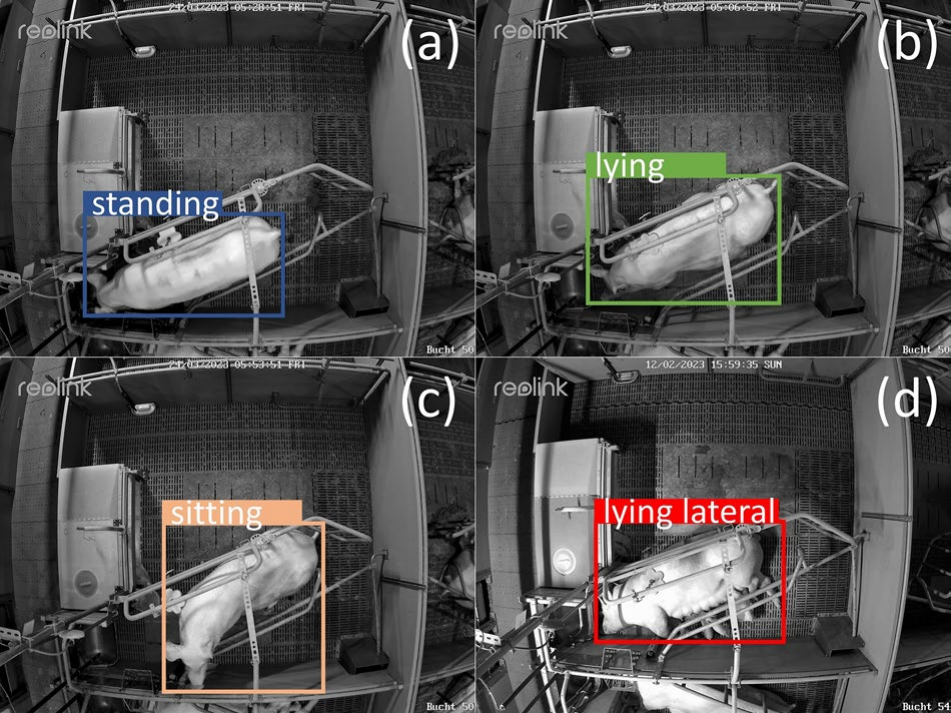
Example images from Env A with the four annotated body posture classes: standing (**a**), lying (**b**), sitting (**c**) and lying lateral (**d**).

**Fig. 2:**
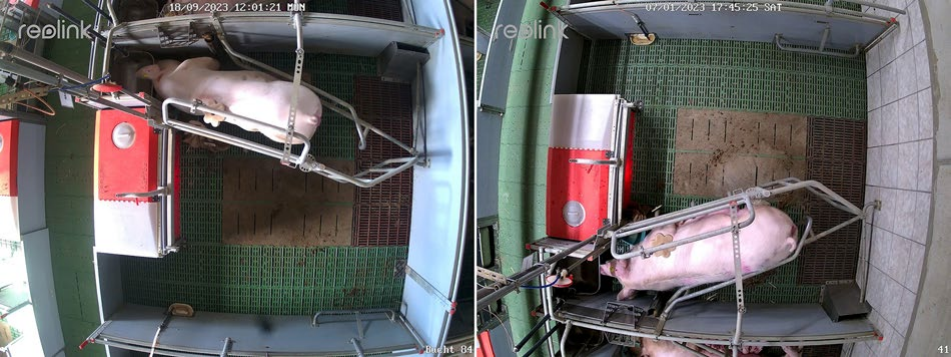
Example images from Env A, which shows transitional postures of sows that have been excluded from the annotation process.

Using clearly defined visual criteria, the body posture of the sow within the bounding box was assessed to determine whether it could be assigned to one of four predefined classes (lying lateral, lying, sitting, standing; Table 2). Classification was based on the sow’s body position relative to the ground and the configuration of its limbs. In particular, special attention was given to distinguishing between visually similar postures such as lying and sitting, based on whether the sow’s body was supported by the front legs or resting along the ventral surface with the legs positioned beneath the body. Representative examples of each posture class are shown in Figure 1. Images in which the posture could not be clearly assigned to one of the defined classes were excluded from the dataset. This primarily included transitional postures between two states. Example images of such transitional postures are illustrated in Figure 2.

**Table 2:** Definition and class mapping of the four postures.

Posture Class	Class integer	Definition
Lying lateral	0	The sow is resting on her side with one shoulder blade touching the ground, the teats exposed.
Lying	1	The sow is resting with her chest and belly touching the ground, her front legs folded beneath the sow’s body, and the teats not exposed.
Sitting	2	The sow is supported by her front legs while the hindquarters are in contact with the ground.
Standing	3	The sow extended all four legs, which are bearing weight. Shoulder, chest and belly are not touching the ground.

Following this procedure, all extracted frames were annotated by two trained observers, with all labels subsequently reviewed and cross-checked. To further assess inter-observer reliability, a subset comprising 10% of the images, balanced across posture classes and environments, was randomly selected and independently compared. The resulting Cohen’s Kappa coefficient was 0.96, indicating a high level of agreement between observers.

Although posture transitions are relevant in behavioral analyses, no separate transition class was included in the annotation scheme. The reason is the highly variable nature of transitional postures, which makes it challenging to define consistent visual patterns suitable for reliable model training. Creating a sufficiently diverse and representative dataset for this class would require substantial annotation effort and still likely result in low classification accuracy. A more practical approach could be to identify potential transition images based on low-confidence predictions across the main posture classes. These samples can then be flagged for further analysis or excluded from downstream processing steps.

To increase usability, the.json file annotations generated with LabelMe were finally converted into the well-known YOLO.txt format. Following the described procedure, a balanced dataset comprising 4,800 images per environment (1,200 images per posture class) was generated. The detailed composition and distribution of the final dataset are described in Data Overview.

### Ethics approval

The dataset was generated exclusively through non-invasive video recordings of sows and piglets under commercial farming conditions. Cameras were installed above the pens and no experimental procedures, interventions, handling, sampling, treatments, or modifications of animal management were performed for the purpose of data collection. Animals were housed, fed, and managed according to the farm’s standard husbandry practices and in compliance with the applicable German animal welfare legislation (TierSchNutztV). The study protocol was reviewed and approved by the responsible Animal Welfare Officers for the respective projects. No biological samples were collected and no animals were euthanized as part of this study.

## Data Records

The SowPostureDS^23^ dataset is publicly available at the opendata@uni-kiel.de research data repository of the Christian-Albrechts-University Kiel, Germany and can be accessed via the following 10.57892/100-316.

The dataset consists of annotated images of sows captured in three different farrowing environments (Env A–C). It includes images recorded under varying conditions, including different housing systems, camera perspectives, lighting conditions, and the presence or absence of piglets. Each image contains a single annotated sow labeled with one of four posture classes (lying lateral, lying, sitting, standing).

The dataset is provided as a compressed archive (“SowPostureDS.zip”) and follows a YOLO-based^26^ object detection structure. The archive contains two main subfolders:

- images/ -.jpg image files
- labels/ - corresponding annotation files in YOLO.txt format

File names included environment-specific prefixes (“ziss”, “digi”, and “inno”) to indicate the source environment. Each image file is paired with a corresponding annotation file sharing the same base filename. All images are provided in.jpg format with a uniform resolution of 1280 × 800 pixels. Each annotation file contains exactly one annotated object (the focal sow). Annotations are stored in YOLO format, where each annotation file accordingly contains a single line with five space-separated values:

• *<class_id><x_center><y_center><bb_width><bb_height>*

Where *class_id* represents the posture class of the sow, *x_center* and *y_center* represent the coordinate center of the corresponding bounding box and *bb_width* and *bb_height* represent the horizontal and vertical bounding box length, respectively. Except for *class_id*, all values are normalized to the range [0, 1] to ensure independence from image resolution. The posture class assignment is stated in Table 1.

## Data Overview

**Fig. 3:**
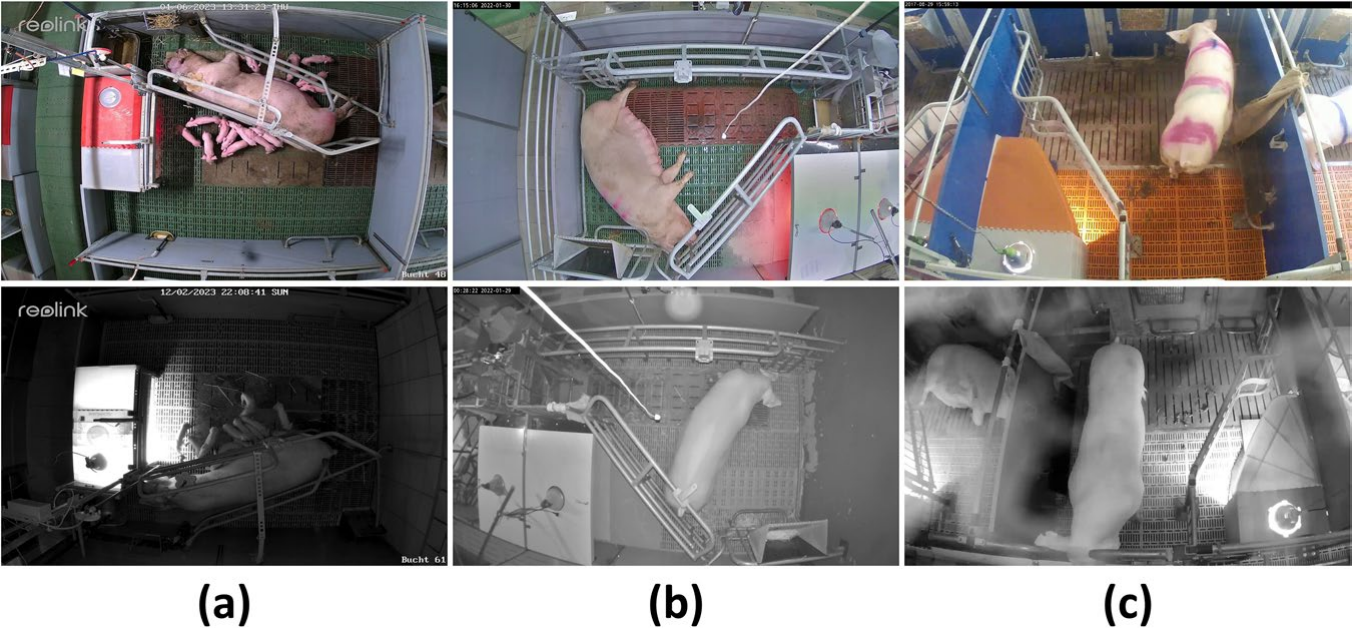
Representative daytime and nighttime images of the three husbandry environments: Env A (**a**), Env B (**b**) and Env C (**c**).

The dataset comprises a total of 14,400 annotated images collected from three different environments (Env A–C). Each environment contributes 4,800 images, with 1,200 images per posture class, resulting in a balanced distribution across classes. A detailed summary of the dataset composition is provided in Table 3. Additionally, Table 4 provides a class-wise breakdown of the number of images per sow for each posture class and environment. Variability in the distribution of images across individual sows reflects differences in study design and data availability between environments. Representative daytime and nighttime images from each housing environment are shown in Figure 3.

**Table 3:** Composition of the dataset across the three environments.

	Env A	Env B	Env C	All
Images	4,800	4,800	4,800	14,400
Images per posture	1,200	1,200	1,200	3,600
Number of videos	98	61	60	219
Number of sows	45	20	9	74
Images per sow	107 (77)	240 (326)	533 (507)	195 (281)
Proportion of Nighttime Images	37.4%	30.2%	21.3%	29.6%

Values for images per sow are reported as mean (SD), with the standard deviation given in parentheses.

**Table 4:** Mean and standard deviations of images per sow per posture class across environments.

Class	Env A	Env B	Env C	All
Lying lateral	27 (23)	109 (197)	240 (212)	59 (118)
Lying	29 (31)	92 (96)	150 (198)	58 (94)
Sitting	31 (33)	100 (203)	150 (129)	61 (112)
Standing	34 (43)	80 (113)	200 (197)	64 (103)

Values are reported as mean (SD), with the standard deviation given in parentheses.

**Fig. 4:**
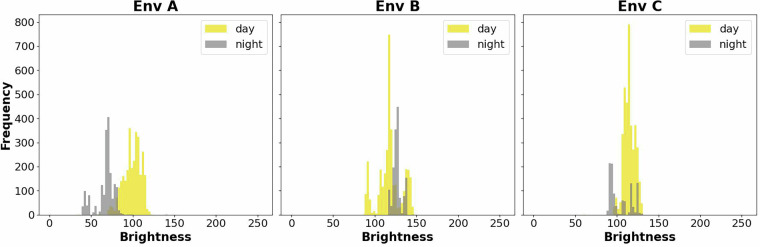
Distribution of average image brightness values for daytime (yellow) and nighttime (gray) images across the three environments.

To characterize the visual properties of the dataset, image-level parameters including brightness, contrast, and color intensity were assessed. These parameters are relevant for computer vision applications^27,28^, as they directly affect feature extraction and model robustness under varying recording conditions. Figure 4 shows the distribution of average image brightness for daytime and nighttime images across the three environments. The distribution illustrates the range of brightness values under different lighting conditions and across environments.

**Fig. 5:**
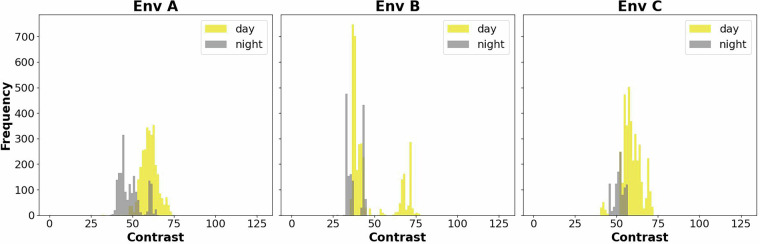
Distribution of average image contrast values across daytime (yellow) and nighttime (gray) images across the three environments. The contrast is calculated as the standard deviation of pixel brightness.

Figure 5 presents the distribution of image contrast values, calculated as the standard deviation of pixel brightness, for daytime and nighttime images in each environment. The distribution illustrates the variability of contrast levels within and between environments and lighting conditions.

**Fig. 6:**
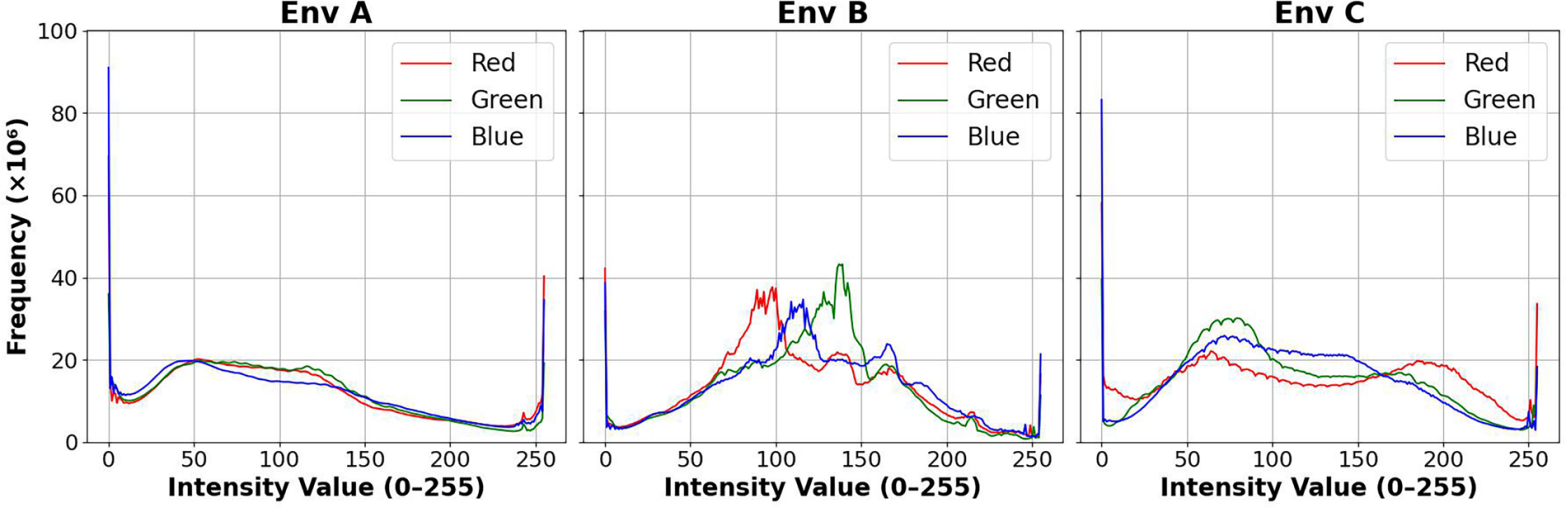
Distribution of pixel intensity values for red, green, and blue channels across the three housing environments. Only daytime images were considered.

Figure 6 shows the distribution of pixel intensity values for the red, green, and blue channels for daytime images across the three environments. The curves illustrate differences in the distribution of color intensities reflecting differences in pen design and lighting conditions between environments. The data included in SowPostureDS were collected over several years in the context of different experimental studies, using varying camera systems and recording configurations. These differences are reflected in the visual characteristics of the dataset.

**Fig. 7:**
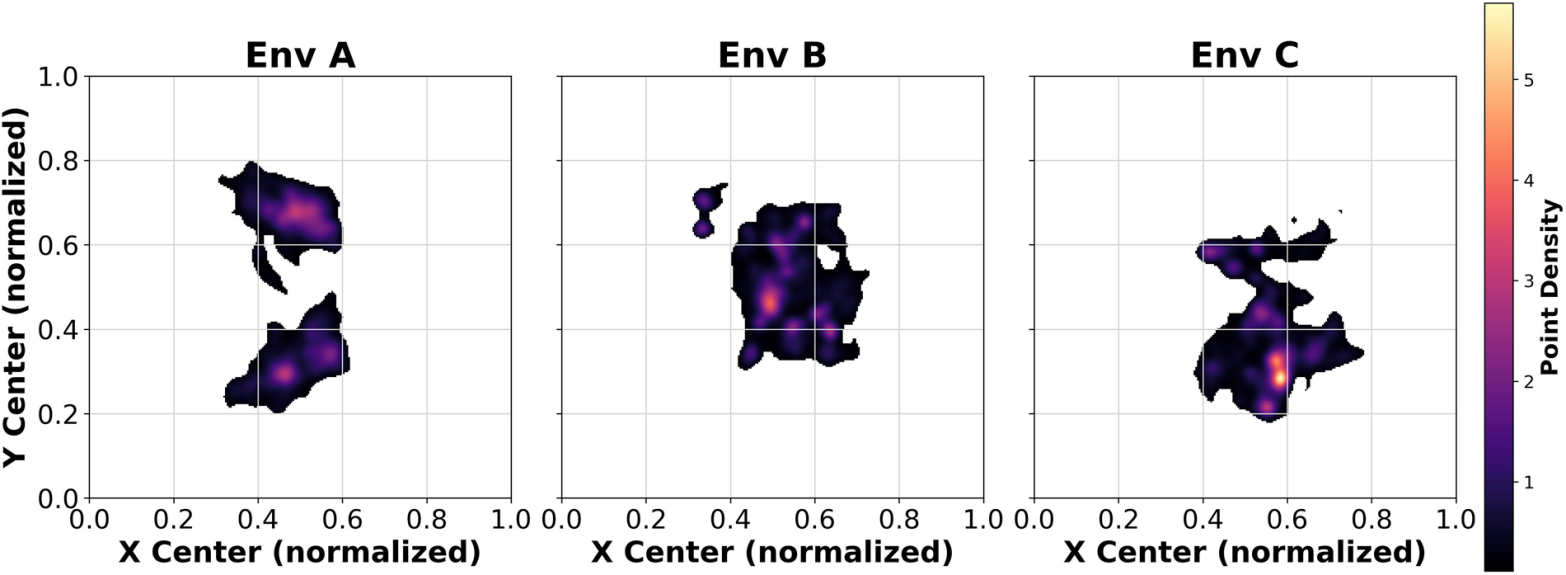
Heat map representation of the spatial distribution of normalized bounding box center points across the three environments.

The spatial distribution of the normalized bounding box center points is shown in Figure 7 using a heat map representation. The figure provides an overview of the positional distribution of the annotated sows, reflecting differences in housing conditions such as confinement and freedom of movement across environments.

## Technical Validation

To assess the practical utility of the proposed SowPostureDS and to demonstrate its potential for training functional CNN models, a use case was designed in which object detection models were trained and evaluated. A particular focus was placed on evaluating the model’s performance when applied to unseen environments, as this is essential for reducing the effort required to adapt models to farm-specific conditions in real-world applications.

As an initial step, it was examined whether a YOLO-based object detection model trained solely on the SowPostureDS could reliably detect and classify sow postures under the diverse conditions represented within the dataset. To this end, training, validation and test subsets were created from the complete dataset while maintaining a balanced class distribution across all three environments. The model was trained on 10,080 images, validated on 2,880 images, and tested on 1,440 images, following a commonly used 7:2:1 splitting ratio for training, validation and testing^29–31^. Training was conducted with the Ultralytics YOLOv11x^26^ architecture for 250 epochs using default augmentation parameters, a batch size of 16 images and an image size of 640.

Model performance was evaluated using standard object detection metrics, including Precision, Recall, and mean Average Precision (mAP)^32,33^, which together provide a comprehensive measure of detection accuracy and robustness. The results for this baseline in-domain evaluation are summarized in Table 5 (Setup I). As can be seen, the model achieved a high detection performance with Precision, Recall and mAP values all above 0.955, demonstrating that SowPostureDS alone provides sufficient variability for accurate posture recognition across the included environments.

**Table 5:** Performance of models trained under different experimental setups, including (I) baseline in-domain evaluation (trained and tested on Env A–C), (II) leave-one-environment-out (LOO) validation, (III) cross-domain evaluation on a new unseen environment (Env D), and (IV) transfer learning.

Training/ Evaluation Setup	Class	Instances	Precision	Recall	mAP50	mAP50-95
(I) Trained on Env A + B + C, tested on Env A + B + C	**All**	**1440**	**0.985**	**0.986**	**0.988**	**0.957**
	Lying lateral	480	0.983	0.992	0.988	0.955
	Lying	480	0.978	0.969	0.982	0.948
	Sitting	480	0.979	0.983	0.988	0.964
	Standing	480	1	1	0.995	0.963
(II) Trained on Env A + B, tested on Env C	**All**	**4800**	**0.422**	**0.493**	**0.375**	**0.263**
	Lying lateral	1200	0.572	0.493	0.571	0.387
	Lying	1200	0.536	0.233	0.336	0.212
	Sitting	1200	0.380	0.378	0.311	0.244
	Standing	1200	0.198	0.869	0.284	0.209
(II) Trained on Env A + C, tested on Env B	**All**	**4800**	**0.402**	**0.622**	**0.407**	**0.318**
	Lying lateral	1200	0.493	0.639	0.532	0.374
	Lying	1200	0.388	0.329	0.290	0.251
	Sitting	1200	0.441	0.751	0.535	0.420
	Standing	1200	0.287	0.770	0.271	0.227
(II) Trained on Env B + C, tested on Env A	**All**	**4800**	**0.415**	**0.551**	**0.406**	**0.319**
	Lying lateral	1200	0.609	0.378	0.450	0.303
	Lying	1200	0.296	0.470	0.265	0.212
	Sitting	1200	0.336	0.650	0.379	0.310
	Standing	1200	0.418	0.706	0.531	0.450
(III) Trained on Env A + B + C, tested on Env D	**All**	**500**	**0.762**	**0.762**	**0.839**	**0.743**
	Lying lateral	125	0.714	0.899	0.919	0.783
	Lying	125	0.722	0.944	0.897	0.810
	Sitting	125	0.958	0.543	0.873	0.802
	Standing	125	0.653	0.662	0.667	0.578
(IV) Trained on Env A + B + C + D (D = 40 images), tested on Env D	**All**	**460**	**0.952**	**0.927**	**0.980**	**0.911**
	Lying lateral	115	0.997	1	0.995	0.913
	Lying	115	0.835	1	0.989	0.928
	Sitting	115	0.976	0.791	0.944	0.900
	Standing	115	1	0.917	0.993	0.904

In the transfer learning setup, 40 images from Env D were used for training, and the remaining 460 images were used for testing.

**Fig. 8:**
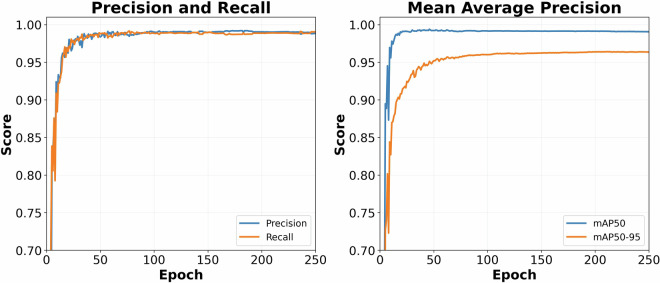
Training history of the baseline YOLOv11x model over 250 epochs, showing the progression of the evaluation metrics (precision, recall, mAP50 and mAP50-95).

In addition, the training process of the baseline model is illustrated in Figure 8, showing the progression of the evaluation metrics over the course of 250 epochs. Precision, recall and mAP rapidly increase during the initial epochs and subsequently stabilize at consistently high levels, indicating stable training behavior under the chosen training setup.

To further assess the robustness of the dataset with respect to domain shifts between environments, a leave-one-environment-out (LOO) validation was performed. In this setup, models were trained on data from two environments and evaluated on the remaining third environment without further adaptation. The results for all LOO combinations are summarized in Table 5 (Setup II). Compared to the baseline in-domain evaluation, a decrease in performance was observed across all configurations. This indicates the presence of domain-specific differences between environments, which affects the direct transferability of models trained on limited subsets of the dataset. The observed differences are consistent across all environment combinations, suggesting that no single environment fully captures the variability present in the complete dataset.

**Fig. 9:**
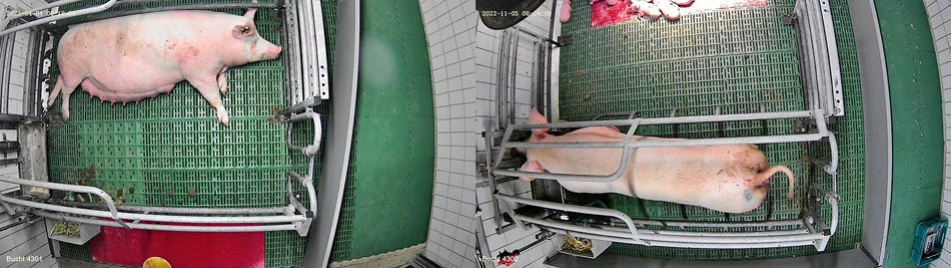
Example images from the unseen test environment (Env D).

Building on the LOO results, the baseline model trained on all three environments was evaluated on a previously unseen environment (Env D) to assess cross-domain performance both without adaptation and in comparison to a transfer learning approach. To this end, an additional dataset (Env D) was created. The videos were recorded at the Chamber of North Rhine-Westphalia in Bad Sassendorf. The dataset consists of 500 annotated images, with 125 images per posture class to ensure balanced representation. Annotation was performed following the procedure described in the Methods section. As SowPostureDS includes sows both with and without piglets, as well as confined and unconfined individuals, the unseen dataset reflects these characteristics. Representative examples are shown in Figure 9. The results for this cross-domain evaluation are summarized in Table 5 (Setup III). Compared to the LOO results, an overall improvement in performance was observed, indicating that training on all available environments increases the robustness of the model when applied to unseen data. However, performance remained below the baseline in-domain evaluation, reflecting the domain shift between the training environments and the unseen target environment.

To evaluate the adaptability of the model to new environments, the transfer learning approach was applied. Therefore, a small subset of 40 images (10 per posture class) from the unseen environment (Env D) was randomly selected and incorporated into the training process. These images were split into training (28 images), validation (8 images), and test (4 images) sets following the established 7:2:1 ratio. Training was then repeated under identical conditions to obtain an updated model. The remaining 460 images from Env D were used for evaluation.

The results for this transfer learning setup are summarized in Table 5 (Setup IV). Compared to the cross-domain evaluation without adaptation (Setup III), a substantial improvement in performance was observed across all metrics. Precision, recall and mAP values increased markedly, approaching the level of the baseline in-domain evaluation. At the class level, performance improvements were observed for all posture classes. The most pronounced improvement was observed for the standing posture, which showed comparatively low performances in the cross-domain evaluation but achieved substantially higher precision and mAP after transfer learning. Similarly, the lying lateral and lying postures showed notable performance gains, reaching high precision and mAP values after transfer learning. The sitting posture showed improved recall compared to the cross-domain evaluation, but remained comparatively lower than the other classes, indicating that certain instances were still more difficult to detect. This is likely due to the visual similarity between sitting and other postures like lying.

**Fig. 10:**
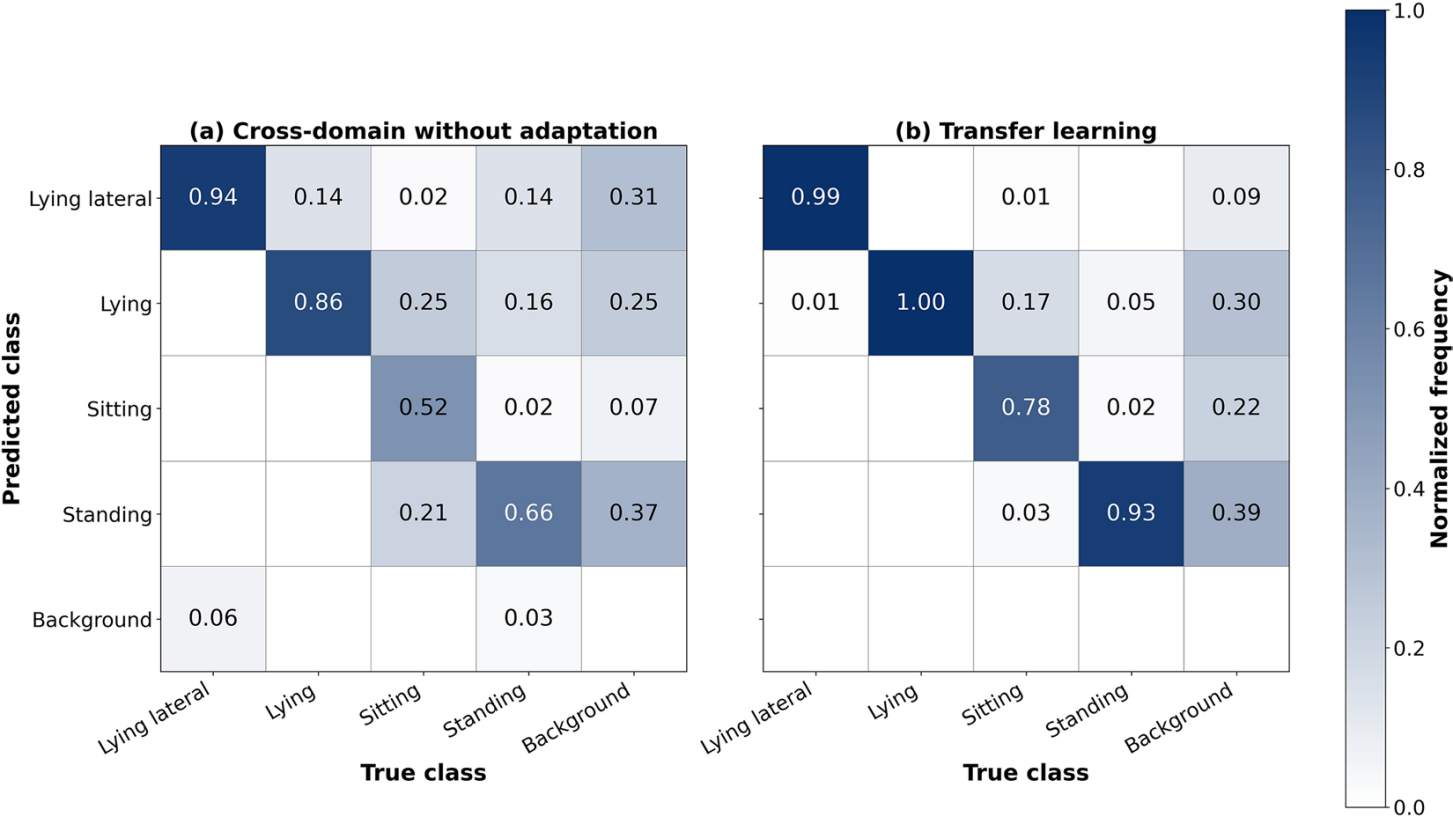
Confusion matrices for predictions on the unseen environment (Env D) for Setup III (without adaptation) and Setup IV (transfer learning). Values are normalized per true class. Background indicates missed detections.

To further analyze the classification performance, confusion matrices for the unseen environment are shown in Figure 10 for (a) the model trained without adaptation (Setup III) and (b) the model after transfer learning (Setup IV). For the model trained without adaptation, the confusion matrix reveals that most misclassifications occur between visually similar posture classes. In particular, sitting is frequently confused with standing and lying, as reflected by the relatively low proportion of correctly classified sitting instances and the noticeable spread across adjacent classes. This may be related to the intermediate nature of the sitting posture, which shares visual characteristics with both lying and standing. Depending on the degree of body support and the viewing angle, sitting postures can resemble partially elevated lying positions or incomplete standing positions, particularly when key body parts such as the front legs are not visible. As a result, sitting can represent a comparatively more challenging posture class for reliable classification. Similarly, standing shows reduced classification accuracy, with a substantial proportion of instances being misclassified as sitting or assigned to the background. In contrast, lying lateral and lying are classified more reliably, although lying still shows minor confusion with sitting and standing.

The potential of the transfer learning approach is reflected in a clear improvement in classification performance across all posture classes. The proportion of correctly classified sitting instances increased substantially, accompanied by a reduction in confusion with standing. Similarly, the classification accuracy of standing improved, with fewer misclassifications into other posture classes. The lying posture also benefits from transfer learning, reaching high classification accuracy in the confusion matrix while maintaining only minor confusion with sitting and standing. Lying lateral, which already showed good performance, remains consistently well classified. Overall, the confusion between posture classes is reduced, and predictions become more concentrated along the diagonal, indicating improved class separability.

In addition to misclassification between posture classes, the confusion matrices also highlight the presence of background predictions, which reflect missed detections. After transfer learning, an increase in background predictions can be observed for some posture classes. This indicates a more refined class representation, where uncertain instances are less frequently assigned to incorrect posture classes and are instead rejected as background. As a result, inter-class misclassifications are reduced, while some ambiguous instances remain undetected. Overall, the confusion matrix analysis demonstrates that classification errors are primarily associated with visually similar or ambiguous postures, particularly for sitting and standing. The observed improvements after transfer learning further indicate that incorporating a small number of representative samples from a new environment can substantially reduce inter-class confusion, while enabling a more robust separation between posture classes in the unseen environment.

These findings are consistent with the quantitative performance metrics reported in Table 5 and highlight that SowPostureDS provides a robust foundation for training posture detection models that can be effectively adapted to new environments using transfer learning.

## Usage Notes

If the intended application requires detecting only the sow located in the center of the image, it is advisable to mask or darken the image borders. Without this adjustment, the models may also detect sows from neighbor pens, which are sometimes partially visible at image margins. For the models trained in the presented use case, the images were not masked, yet good performances were achieved. However, applying such processing could potentially lead to further slight improvements in detection accuracy by reducing false positives from neighboring animals.

## Data Availability

The dataset generated in the course of this study is publicly available at the opendata@uni-kiel.de research data repository of the Christian-Albrechts-University Kiel and can be accessed via the following 10.57892/100-316. The additional images from the unseen environment (Env D) used for technical validation are not part of the published dataset due to data usage restrictions and are therefore not publicly available.

## Code availability

No custom code was used for data processing. All procedures were performed using standard, publicly available software and libraries.
